# Intra-aortic band impairs transapical device implantation in a pig: a case report

**DOI:** 10.1186/s12917-023-03781-3

**Published:** 2023-10-18

**Authors:** Florian Meissner, Michelle Costa Galbas, Johannes Dinkelaker, Heidi Cristina Schmitz, Hendrik Straky, Johanna Reuter, Martin Czerny, Wolfgang Bothe

**Affiliations:** 1 Department of Cardiovascular Surgery, University Heart Center Freiburg, Hugstetter Strasse 55, 79106 Freiburg, Germany; 2 Faculty of Medicine, University of Freiburg, Freiburg, Germany; 3 Center for Experimental Models and Transgenic Service, Faculty of Medicine, University of Freiburg, Stefan-Meier-Strasse 17, 79104 Freiburg, Germany

**Keywords:** Congenital anomalies, Intra-aortic band, Animal testing, Pig, Endovascular procedures, Left ventricular assist device

## Abstract

**Background:**

Anatomic anomalies in the ascending aorta may impair the implantation and testing of cardiovascular devices in humans and animal models.

**Case presentation:**

We present the rare case of an intra-aortic band in a German Landrace pig. During terminal animal testing, the band hindered the implantation of a left ventricular assist device (LVAD) with transventricular outflow graft across the aortic valve. After lower partial sternotomy, epicardial echocardiography displayed an intraluminal echogenic structure at the sinotubular junction causing unspecific flow turbulences. Under cardiopulmonary bypass, coring of the left ventricular apex was performed. Due to strong resistance in the proximal aorta, accurate positioning of the transventricular LVAD outflow graft was impossible. After euthanasia, necropsy revealed a fibrous band located at the sinotubular junction, dividing the lumen of the ascending aorta.

**Conclusions:**

The occurrence of an intra-aortic band represents an extremely rare case of a most likely congenital anomaly. Awareness of such anomalies is important for planning and performing animal testing. Perioperative echocardiography may help to either remove such anomalies or allow discontinuing the procedure prior to device implantation.

## Background

Anatomical variations may affect in vivo studies in large animal models including medical device testing. In case of severe secondary complications, acute termination of animal testing might be indicated. In general, endovascular procedures require optimal vascular access for the introduction and delivery of guide wires, catheters and medical devices including stent grafts and transcatheter implanted heart valves. We present the rare case of an anomalous band in the proximal ascending aorta (AAo) impairing medical device implantation in a pig. Awareness of such anomalies and knowledge about their diagnosis and treatment during animal testing might support surgeons and cardiologists in decision-making contributing to an improved outcome. Overall, this case highlights the importance of pre- and intraoperative imaging for safe and successful animal testing.

## Case presentation

This case was part of a series of acute animal testing to assess a modified left ventricle assist device (LVAD, HeartMate 3, Abbott, Abbott Park, IL) in pigs. All experiments were approved by the local ethics committee (Regierungspräsidium Freiburg, Germany, approval number 35-9185.81/G-22/006). The research was conducted in accordance with the German animal protection law (TierSchG), the European Convention for the Protection of Vertebrate Animals used for Experimental and other Scientific Purposes, and the ARRIVE guidelines 2.0 [1, 2]. The porcine model was chosen due to the similarities with human cardiovascular anatomy and physiology.

**Fig. 1:**
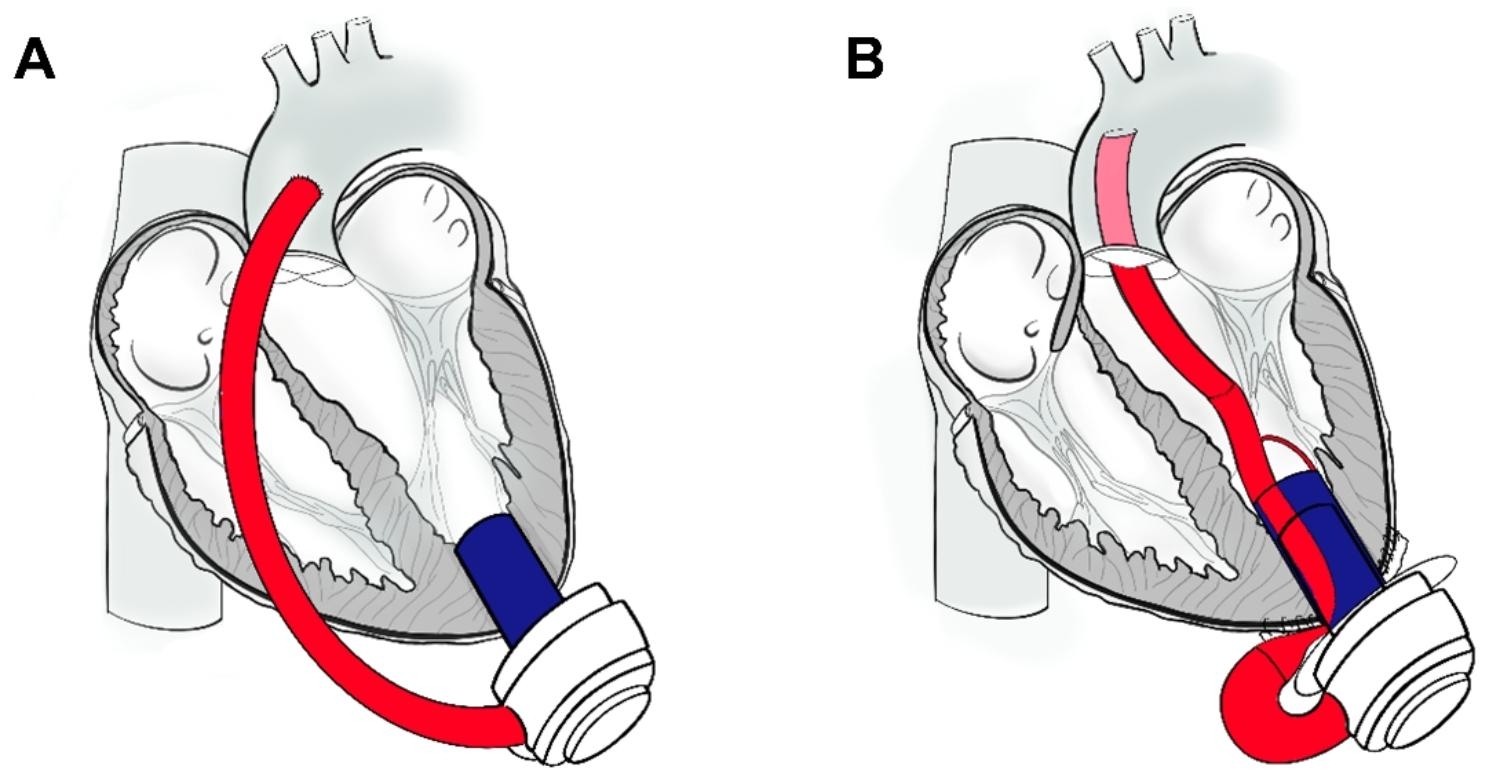
Left ventricular assist device (LVAD) with (A) conventional outflow graft as well as (B) LVAD accessory with transventricular outflow graft (figures representing human anatomy). Adapted from Meissner et al. [3]

Mechanical circulatory support devices such as LVAD systems allow left ventricular (LV) unloading in case of severe heart failure. Conventional LVADs comprise an extracardiac outflow graft (OG), which directs the blood from the transapical inflow towards the AAo (Fig. 1A). We develop an LVAD accessory redirecting the blood through a transventricular OG across the aortic valve (AV) (Fig. 1B) [3]. Applying the LVAD accessory allows less invasive LVAD implantation via a single transapical approach [4].

This case refers to a female German Landrace pig (88 kg), which showed no signs of disease prior to surgery. The pig was accommodated in the experimental animal facility of the University of Freiburg, fed a standard pellet chow (20 g/kg), and had access to water ad libitum. The animal was kept under controlled environmental conditions at 20 °C, 75 ± 5% humidity, and a 13/11-hour light/dark cycle. Prior to surgery, no imaging was performed, neither for surgical planning nor to rule out anatomic variations.

Pre-medication was induced with Ketamine (20 mg/kg IM) and Midazolam (0.5 mg/kg IM). After initial sedation, anesthesia was induced with propofol (2‒4 mg/kg IV) and vecuronium (0.2 mg/kg IV), and maintained, after endotracheal intubation, with propofol (10‒15 mg/kg IV), fentanyl (5‒10 µg/kg IV) and vecuronium (0.2‒0.4 mg/kg IV). Cefazolin (2 g IV) and Ringer’s solution (10 mL/kg/h) were administered as prophylactic antibiotic and fluid substitute, respectively. For pressure-controlled ventilation, F_i_O_2_ (20‒40%), tidal volume (6‒8 mL/kg), initial PEEP (5‒8 mbar) and respiratory frequency (12‒18/min) were adjusted to maintain physiological conditions. Standard perioperative monitoring was applied and the animal was placed in supine position.

**Fig. 2:**
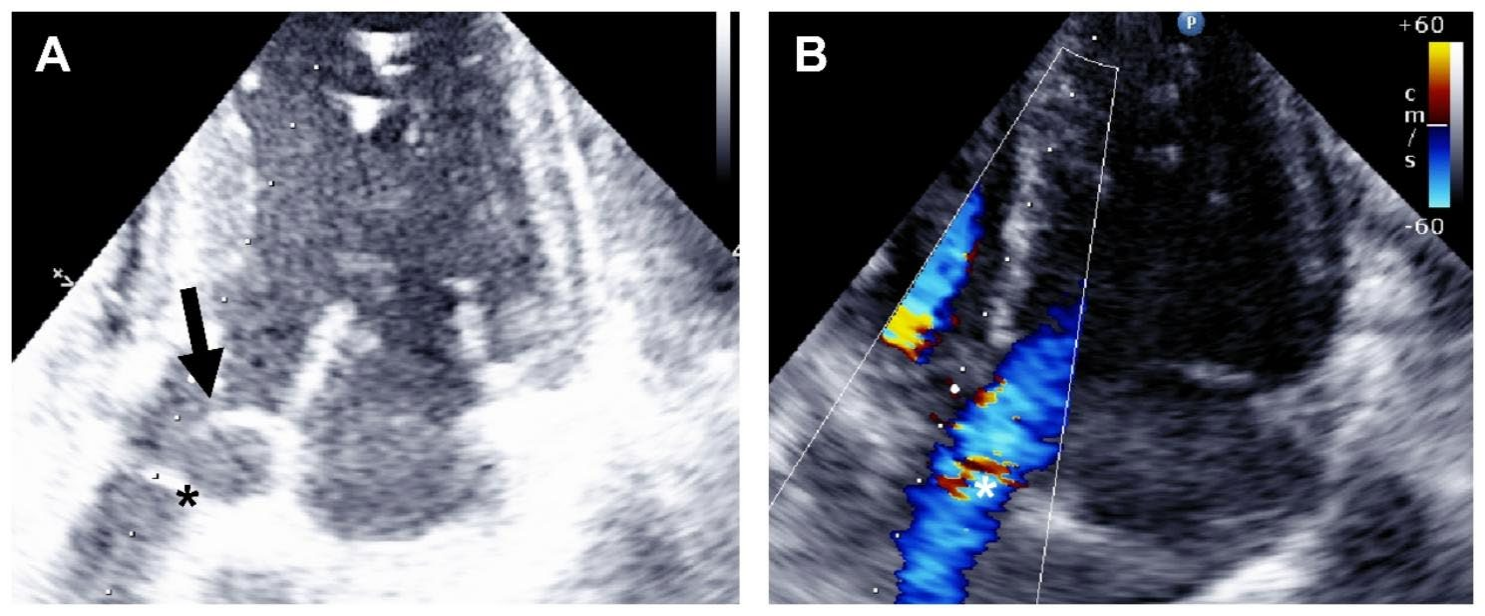
Intra-aortic band in (**A**) 2D echocardiography and (**B**) with color flow Doppler. (**A**) In 2D echocardiography, an intra-aortic hyperechoic structure (*) distal to the aortic valve (arrow) was found at the level of the sinotubular junction. (**B**) Color flow Doppler displayed unspecific turbulences around this structure

For balloon-assisted delivery of the transventricular OG, an introducer sheath was inserted into the right common carotid artery. For CPB, the right femoral vein and artery were cannulated. After lower partial sternotomy, epicardial echocardiography revealed normal heart size and function. An anomalous intra-aortic hyperechoic structure was found at the level of the sinotubular junction (STJ) (Fig. 2A). Color flow Doppler indicated unspecific flow turbulences nearby this structure (Fig. 2B).

**Fig. 3:**
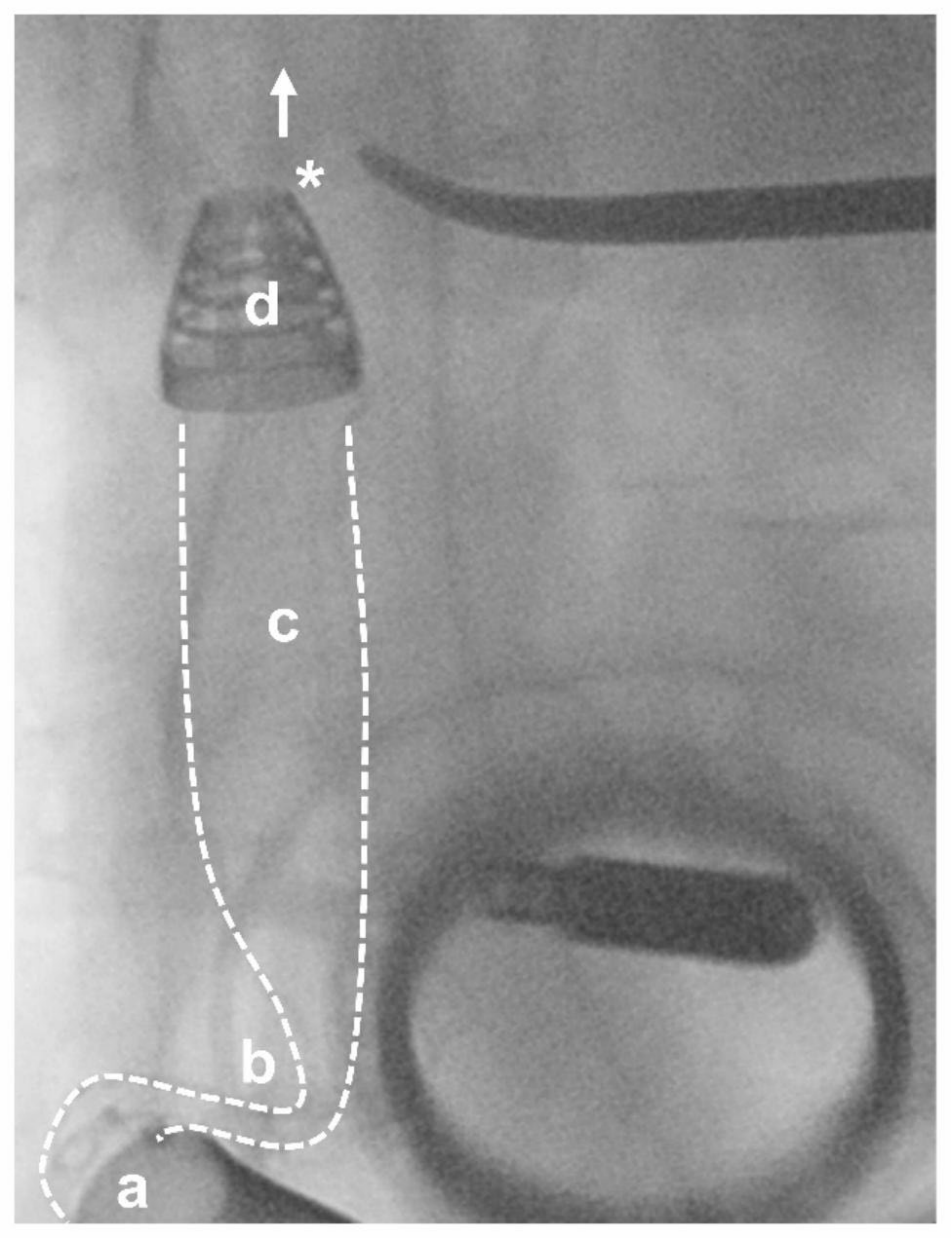
Fluoroscopy of the implantation site with outflow graft kinking. Distal to the outlet of the LVAD accessory (**a**), kinking (**b**) of the proximal transventricular outflow graft (**c**) was seen. The outflow tip (**d**) could not be positioned further into the ascending aorta (arrow) due to the intra-aortic band (*)

Subsequent to the insertion of a guidewire through the introducer sheath in the right common carotid artery, a balloon catheter was retrogradely delivered into the LV. After transapical attachment of the LVAD sewing ring and CPB initiation, surgical coring of the LV apex was performed (balloon dilatation results in myocardial tearing). Thereafter, the balloon catheter was retracted from the LV through the apical coring site and positioned into the OG. After balloon inflation within the OG, the catheter was partially withdrawn from the right carotid artery. Thereby, the OG loaded on the balloon was delivered from the apical coring site through the LV across the AV. Resistance in the proximal AAo hindered further balloon withdrawal and accurate OG positioning. Fluoroscopy revealed OG kinking and malpositioning of the OG tip just above the AV (Fig. 3).

After balloon deflation and removal, the LVAD was activated and the pig weaned from CPB. Within minutes, the pig developed ventricular fibrillation and had to be defibrillated several times. To prevent the animal from further suffering, euthanasia was performed, after propofol overdose, by administration of potassium chloride (2 mmol/kg IV). An LVAD ramp to assess the hemodynamic impact of changes in the rotational speed, had to be cancelled.

**Fig. 4:**
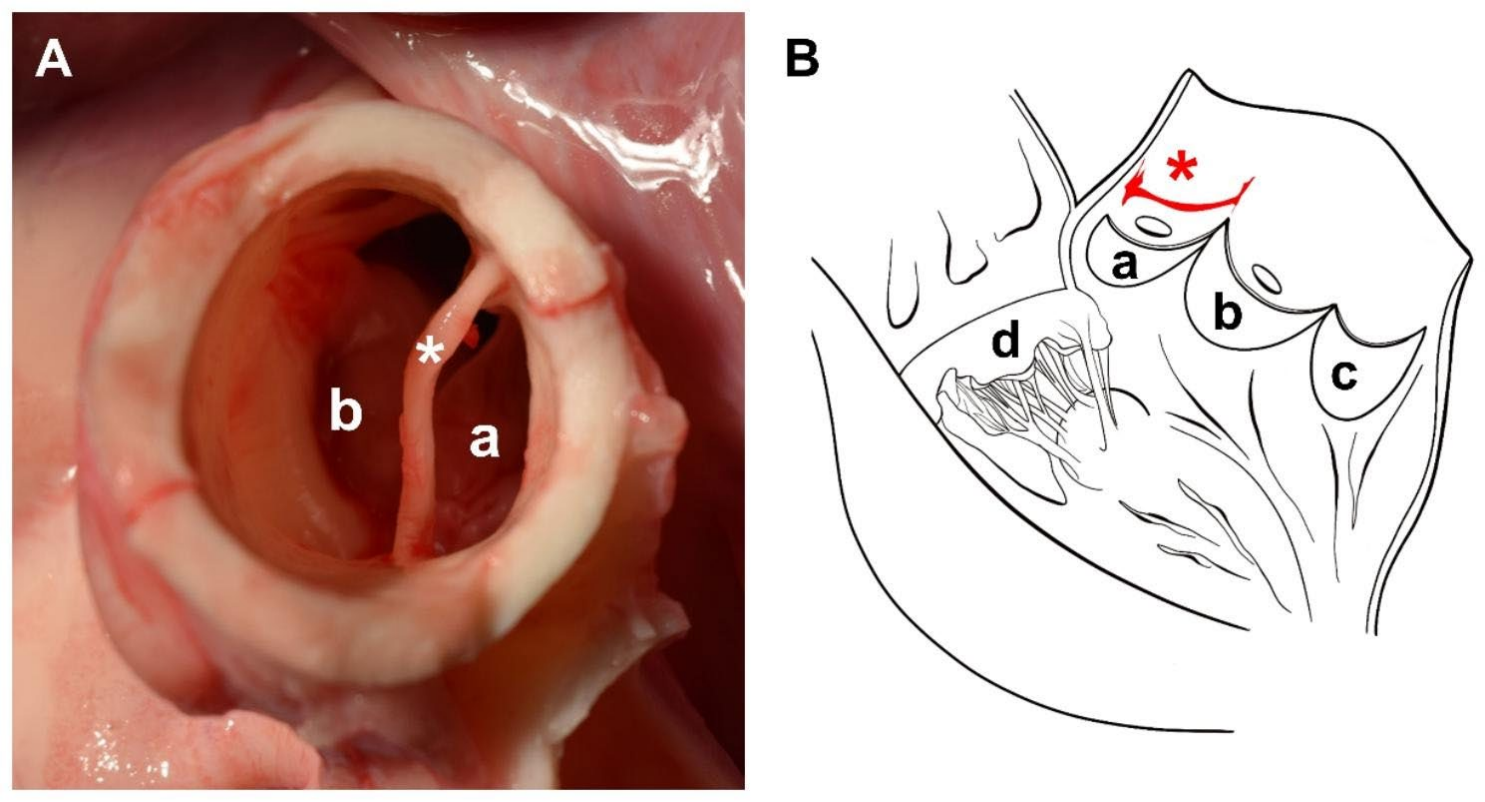
(**A**) Post mortem examination of the aortic valve and the ascending aorta, and (**B**) schematic representation of the band’s position found after opening the heart. (**A**) The intra-aortic band (*) was found at the sinotubular junction. The left coronary cusp (**a**) and the right coronary ostium **(b)** are shown below the band. (**B**) The band (*) was located between the commissures of the left coronary cusp (**a**). Moreover, the right (**b**) and non-coronary aortic cusp (**c**) and the mitral valve (**d**) are depicted

After heart explantation, an intra-aortic band was found at the STJ (Fig. 4A). It was oriented transversally between the commissures of the left coronary cusp (Fig. 4B). The band was 1.8 cm in length and up to 2.7 mm thick. It divided the lumen of the proximal AAo into a narrow (1.7 × 0.8 cm, 0.7 cm^2^) and a wide passage (2.3 × 1.3 cm, 2.9 cm^2^). The band appeared to be fibroelastic applying no relevant tension on the AAo wall.

## Discussion and conclusions

We describe the rare case of a fibrous band dividing the lumen of the proximal AAo, which was found during LVAD implantation in a pig. The intra-aortic band impaired the delivery of the transventricular device OG into the AAo using a balloon catheter. We assume that the wire guiding the balloon catheter was inserted through the narrow passage of the divided AAo, which appeared to be too small for balloon retraction. Due to persisting ventricular fibrillation and later on asystole, the animal testing had to be terminated early without functional LVAD assessment.

What can be learn from this case? First of all, we should be aware of aortic anomalies impairing medical device testing (e.g., LVAD or stent implantation, transcatheter AV replacement). Although such cases are extremely rare and impair only few procedures, they affect the reproducibility and reliability of these studies. Such findings might require acute test termination, reducing the study’s power in small sample sizes and causing additional costs.

Moreover, perioperative imaging might help to detect intra-aortic anomalies and support optimal animal selection. However, depending on the anomaly’s location and size, its detection might be difficult. In pigs, transthoracic echocardiography is rather challenging for anatomical reasons (e.g., prominent rib cage, overlying lungs), and transesophagael echocardiography allows only incomplete depiction of the distal AAo and the aortic arch. In contrast, intraoperative epicardial (as applied in this case) and intra-aortic echocardiography allow excellent views. In specific cases (e.g., porcine-to-human xenotransplantation), preoperative computed tomography should be performed to exclude anatomical anomalies and relevant size mismatches [5].

Depending on the band’s location and size as well as the time of its detection, there are different options to proceed. In case of incidental preoperative diagnosis and a high probability for test impairment, a healthy animal should be selected instead. In case of intraoperative diagnosis, the veterinarian, surgeon or cardiologist has to decide whether the anatomic anomaly affects the intended procedure (e.g., LVAD or stent implantation) or is clinically relevant, potentially affecting long-term testing (e.g., turbulences resulting in thrombus formation, high shear stresses favoring von Willebrand factor degradation). If applicable, there are several interventional (e.g., loop snare, endovascular bioptome) and surgical treatment options (e.g., open resection, aortic replacement). In any case, we do not recommend using a balloon catheter for either dilating the band, tearing it off (analog to Rashkind balloon procedure) or pushing it towards the aortic wall. Increased tensile forces on the intra-aortic band may favor severe injuries to the AAo wall and AV, resulting in secondary aortic dissection and/or regurgitation. In rare cases, increased tensile forces may also lead to a damage of the balloon catheter.

Since we did not perform any histopathologic examination, detailed information regarding the band’s constitution are missing. In accordance with its macroscopic appearance, elastic behavior and previous cases, the band was most likely composed of elastic connective tissues, such as collagen, covered by an endothelial layer [6, 7]. In one case report, the elastic tissue emerged into the elastic tissue of the aortic wall [8]. Overall, cases of intra-aortic bands associated with the AV have been described in humans with congenital bicuspid AV. Nakamura et al. reported two cases of an abnormal fibrous band between the center of the conjoined cusp and the aortic wall, leading to aortic regurgitation and stenosis [9]. Intravascular bands are found mainly in the proximal AAo, hypothetically as supportive structures to the AV [10]. Bonde et al. described one case of a band within the proximal aortic arch [7]. Rarely ever, such bands are found in the abdominal aorta [10]. Although the exact etiology remains unclear, the case of a stillborn having a similar intra-aortic band indicates a congenital genesis. Regardless of animal testing, the occurrence of an intra-aortic band does not seem to be life threating, but might create a heart murmur and lead to rather negligible flow turbulences [8].

In comparison to bicuspid AV, valvular fenestration and deviations, an intra-aortic bands represents an extremely rare case of a most likely congenital anomaly [8]. It is important to be aware of such anomalies to ensure safe and successful animal testing, to manage secondary complications and to apply pigs as donors for biologic implants. To our best knowledge, this is the first case of an intra-aortic band reported in a pig model.

## Data Availability

The data supporting this article’s findings are available from the corresponding author, Florian Meissner [florian.meissner@uniklinik-freiburg.de].

## Abbreviations

AAo: Ascending aorta
AV: Aortic valve
CPB: Cardiopulmonary bypass
F_i_O_2_: Fraction of inspired oxygen
IM: Intramuscular
IV: Intravenous
LV: Left ventricle
LVAD: Left ventricular assist device
OG: Outflow graft
PEEP: Positive end-expiratory pressure
STJ: Sinotubular junction
